# Determination of the Dielectric Properties of Storage Materials for Exhaust Gas Aftertreatment Using the Microwave Cavity Perturbation Method

**DOI:** 10.3390/s20216024

**Published:** 2020-10-23

**Authors:** Carsten Steiner, Stefanie Walter, Vladimir Malashchuk, Gunter Hagen, Iurii Kogut, Holger Fritze, Ralf Moos

**Affiliations:** 1 Bayreuth Engine Research Center (BERC), Department of Functional Materials, University of Bayreuth, 95440 Bayreuth, Germany; Stefanie.Walter@uni-bayreuth.de (S.W.); vladimir.malashchuk@uni-bayreuth.de (V.M.); gunter.hagen@uni-bayreuth.de (G.H.); ralf.moos@uni-bayreuth.de (R.M.); 2 Institute of Energy Research and Physical Technologies, Clausthal University of Technology, 38640 Goslar, Germany; iurii.kogut@tu-clausthal.de (I.K.); holger.fritze@tu-clausthal.de (H.F.)

**Keywords:** microwave cavity perturbation, radio frequency, resonant frequency, quality factor, dielectric properties, depolarization, powders, ceria, exhaust gas aftertreatment, on-board diagnosis (OBD)

## Abstract

Recently, a laboratory setup for microwave-based characterization of powder samples at elevated temperatures and different gas atmospheres was presented. The setup is particularly interesting for *operando* investigations on typical materials for exhaust gas aftertreatment. By using the microwave cavity perturbation method, where the powder is placed inside a cavity resonator, the change of the resonant properties provides information about changes in the dielectric properties of the sample. However, determining the exact complex permittivity of the powder samples is not simple. Up to now, a simplified microwave cavity perturbation theory had been applied to estimate the bulk properties of the powders. In this study, an extended approach is presented which allows to determine the dielectric properties of the powder materials more correctly. It accounts for the electric field distribution in the resonator, the depolarization of the sample and the effect of the powder filling. The individual method combines findings from simulations and recognized analytical approaches and can be used for investigations on a wide range of materials and sample geometries. This work provides a more accurate evaluation of the dielectric powder properties and has the potential to enhance the understanding of the microwave behavior of storage materials for exhaust gas aftertreatment, especially with regard to the application of microwave-based catalyst state diagnosis.

## 1. Introduction

In order to meet the legal emission standards, customized automotive exhaust gas aftertreatment systems are required. They are still subject of research today. New concepts have been presented in recent years with the aim of further reduction of emissions. One of these technologies is the radio frequency (RF)-based state diagnosis of automotive catalytic converters and filter systems. Using the microwave cavity perturbation (MCP) method, the loading state of an exhaust gas aftertreatment components can be measured *operando*, i.e., during operation, without contacting electrodes [1,2,3]. In this method, the catalyst housing serves as a cavity resonator, in which standing electromagnetic waves are excited at discrete frequencies via coupling elements (also designated as antennas). The properties of these so-called resonant modes depend on the dielectric properties of the installed catalyst or filter and therefore change with the loading state of the exhaust gas aftertreatment component.

A number of studies have already shown that the method can be successfully applied to many common types of catalysts and particulate filters. In case of gasoline engines, the RF approach can be used to determine the oxygen storage state of three-way catalytic converters (TWC) [4,5,6,7,8]. For coated gasoline particulate filters, both the degree of oxidation and the soot load can be evaluated [9,10]. In addition, the system has already been successfully applied to diesel vehicles. The ammonia load of a SCR (selective catalytic reduction) catalyst [11,12,13], the nitrogen oxide (NO*_x_*) storage of a lean NO*_x_* trap [14,15], and the soot load of a diesel particulate filter (DPF) [16,17] can be evaluated with the RF system.

These microwave-based systems are particularly useful for real-world applications. In this configuration, the catalyst or filter component always fills the largest part of the resonator volume. This setup is therefore not suitable for determining the exact dielectric properties of exhaust gas aftertreatment components and materials due to the large perturbation inside the cavity resonator. In order to optimize the technical application and to learn more about the radio frequency characteristics of exhaust systems, a cylindrical microwave test bench was recently presented, which allows applying more defined conditions [18]. The laboratory setup was developed to investigate small samples, such as the pure storage component of a catalyst or a crushed catalyst, at typical operating conditions. With this setup, H-form and Cu-exchanged ZSM-5 powders for SCR applications could be characterized dielectrically in detail [19,20]. Recently, a study was published that deals with the RF response of ceria (CeO_2_), the oxygen storage component of the TWC [21]. The effect of Pt on the reducibility of CeO_2_ at low temperatures was also confirmed with the microwave technique.

The specially developed laboratory test setup enables a much more precise examination of powder samples for exhaust gas aftertreatment and provides fundamental knowledge about their RF characteristics. Nevertheless, the exact calculation of material properties for the investigated samples is not trivial, since some conditions of the classical microwave cavity perturbation theory (MCPT) are not completely met. Previous publications use a simplified approach and deliver at least basic statements about the relative change of the polarization *ε*_1_ and the dielectric loss *ε*_2_ during operation. However, exact values cannot be determined with this method.

In this study, an approach is presented that allows the exact calculation of the dielectric sample properties. In the first section, the existing resonator is briefly introduced, and the measurement method is evaluated according to the criteria of the simplified MCP theory. Subsequently, approaches to describe the essential effects in the resonator beyond the simplified MCPT are presented. The work combines accepted analytical ideas with results from electric field simulations of the existing setup. The focus of this work is on the consideration of the electric field distribution in the resonator as well as on the depolarization of the sample and the calculation of material properties from the effective bulk properties of the powder. The presented approach is verified with first measurements on a ceria sample by comparison with literature findings. Finally, the accuracy of the calculation method, alternative approaches and the transferability of the solution to other typical materials for exhaust aftertreatment are discussed.

## 2. Laboratory Setup, Simulation Model and MCP-Fundamentals

### 2.1. Resonator Setup

**Figure 1:**
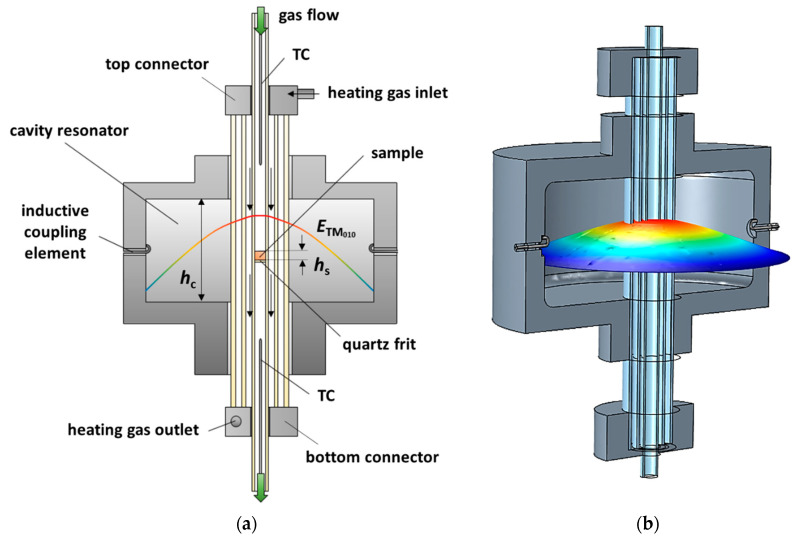
Illustration of the resonator setup for MCP measurements with the electric field (qualitatively) of the TM_010_ mode: (**a**) schematic sectional view of the setup; (**b**) simulation model in COMSOL Multiphysics^®^ 5.5.

Figure 1a shows the schematic sectional view of the resonator geometry and Figure 1b demonstrates the three-dimensional simulation model. The cylindrical cavity resonator (Ø 184 mm, height *h*_c_ 80 mm) is made of aluminum. Several quartz glass tubes are inserted along the resonator axis. The sample with a height *h*_s_ is located in the center of the resonator cavity on a porous quartz glass frit in the inner tube (Ø 10 mm) and can be flushed vertically with different process gases. The inner sample tube is encased by a double tube (Ø 20 mm and Ø 38 mm). The space between the double tube is evacuated to minimize thermal losses. Around the inner sample tube, the sample is heated indirectly by flowing heating gas (hot air), which allows sample temperatures of up to 600 °C. To measure the sample temperature, the NiCr-Ni thermocouples (TC) are inserted into the sample tube from above and below the resonator. Thus, the sample temperature is determined by taking the arithmetic mean of both temperatures [18]. Additionally, the thermocouples penetrate the sample tube only so far that they do not affect the RF measurement. A circulated water cooling is used to maintain the room temperature of the aluminum resonator during the operation. Further details on the design (sealing system, temperature distribution, thermal considerations, etc.) and more technical specifications can be found in [18].

Electromagnetic energy provided by a vector network analyzer (Anritsu ShockLine MS46322b) is coupled inductively into the cavity resonator via two loop antennas. At certain frequencies, standing waves, so-called resonant modes, propagate within the resonator cavity. The resonator is designed to dielectrically characterize samples with the TM_0*n*0_ modes (*f*_TM_010__ = 1.18 GHz, *f*_TM_020__ = 2.62 GHz, *f*_TM_030__ = 4.19 GHz). Usually, these modes have a constant *E*-field maximum along the resonator axis. The sample is, therefore, always located in the electric field maximum, the area with the highest RF sensitivity. The field distribution of the TM_010_ fundamental mode, which has exactly one field maximum along the resonator axis, is shown qualitatively in Figure 1a,b.

In the following, a comprehensive simulation model (COMSOL Multiphysics^®^ 5.5), reflecting the detailed cavity resonator design is used as well (Figure 1b). Implemented are the resonator housing made of aluminum (conductivity *σ*_Al_ = 3.8 10^7^ S/m), the quartz glass tubes (dielectric constant *ε*_r,quartz_’ = 4.35, no losses), and antennas as well as the top and bottom connectors made of stainless steel (*σ*_Steel_ = 4.0⋅10^6^ S/m). For air as the filling medium, a dielectric constant *ε*_r,air_’ = 1 and magnetic constant *µ*_r,air_’ = 1 were assumed. Calculations for the electrical field distributions were carried out by a modal analysis (eigenvalue problem).

### 2.2. Microwave Cavity Perturbation Theory

Using the MCP method, the dielectric properties of the sample are determined by means of the differential measurement between the empty and a sample-filled resonator. The sample has the complex permittivity *ε* [22,23]:

(1) $$\varepsilon =\varepsilon^{\prime }-j\varepsilon^{\prime \prime }={\varepsilon_{r}}^{\prime }\varepsilon_{0}-j({\varepsilon_{r}}^{\prime \prime }\varepsilon_{0}+\frac{\sigma }{\omega }),$$

with the permittivity $\varepsilon^{\prime }$ and the dielectric loss $\varepsilon^{\prime \prime }$. The permittivity $\varepsilon^{\prime }$ is the product of the more commonly used relative dielectric constant ${\varepsilon_{r}}^{\prime }$ of the sample and the permittivity of the vacuum *ε*_0_ = 8.854 ⋅ 10^−12^ As/(Vm). The dielectric losses $\varepsilon^{\prime \prime }$ include the (relative) losses ${\varepsilon_{r}}^{\prime \prime }$ due to the sample polarization in the alternating electromagnetic field as well as the ohmic losses due to the electric conductivity *σ*. The ohmic loss is a function of the circular frequency *ω* = 2π*f* of the electromagnetic wave. The change in resonant properties of the cavity resonator due to the insertion of a sample can be derived from Maxwell’s equations. Generally, it has the from [22,23,24,25,26,27]:

(2) $$\left(\frac{f_{0}-f_{S}}{f_{0}}\right)+\frac{j}{2}\left(\frac{1}{Q_{S}}-\frac{1}{Q_{0}}\right)\;=\frac{{∭}_{V_{C}}[(\varepsilon -\varepsilon_{0}{)E}_{1}\cdot E_{0}^{\;*}+(\mu -\mu_{0}{)H}_{1}\cdot H_{0}^{\;*}]dV}{{∭}_{V_{C}}{(\varepsilon }_{0}E_{1}\cdot E_{0}^{\;*}+\mu_{0}H_{1}\cdot H_{0}^{\;*})\;dV}$$

with the cavity volume *V*_C_, the resonant frequencies *f*_0_ and *f*_S_, the (unloaded) quality factors *Q*_0_ and *Q*_S_ of the empty and filled resonator, the electric and magnetic fields *E*_0_ and *H*_0_ of the empty resonator, their complex conjugated fields $E_{0}^{\;*}$, $H_{0}^{\;*}$ and the corresponding fields *E*_1_ and *H*_1_ of the filled resonator. For the volume outside of the sample the dielectric permittivity *ε*_0_ and the magnetic permeability *µ*_0_ of vacuum are assumed. In many cases this relation can be simplified. According to the classical MCP theory, the following correlation applies to the resonant properties of the TM_0n0_ modes of cylindrical resonators and the dielectric properties of an inserted sample, located in the central electric field maximum [22,23]:

(3) $$\frac{\Delta f}{f_{0}}=\frac{{(f}_{0}-f_{S})}{f_{0}}\;={\;(\varepsilon }_{r}{}^{\prime }-1)\;\frac{V_{S}}{{2V}_{\mathrm{eff}}}$$

(4) $$\Delta \left(\frac{1}{Q}\right)\;=\;\frac{1}{Q_{S}}-\frac{1}{Q_{0}}\;={{\;\varepsilon }_{r}}^{\prime \prime }\;\frac{V_{S}}{V_{\mathrm{eff}}}$$

with the sample volume *V*_S_ and effective volume *V*_eff_ of the resonant mode (also denominated as mode volume). The mode volume considers the electric field distribution inside the cavity and corresponds to a kind of (inverse) “RF sensitivity” for the measurement method [22,23]. The smaller *V*_eff_, the higher is the change in the resonant parameters due to the insertion of a sample. The size of *V*_eff_ for ideal cylindrical resonators can be calculated [22,23,25]:

(5) $$V_{\mathrm{eff}}={J_{1}}^{2}{(p}_{0n}{)\;V}_{\mathrm{res}}$$

with the Bessel function *J*_1_ of the 1st kind and 1st order and the *n*-th zero *p*_0*n*_ of the Bessel function of the 1st kind and 0th order for the TM_0*n*0_ mode. In case of the TM_010_ mode *V*_eff_ is 26.95% of *V*_res_. In such cases, the dielectric properties of the sample can be calculated easily from the changes in the resonant properties. The validity of this approach is, however, subject to a number of basic pre-conditions that are particularly crucial for the investigation of powders for exhaust aftertreatment [22,23,24,25,26,27,28,29,30,31,32,33]:

- The sample volume is small compared to the resonator volume.
- The sample height and the resonator height are identical (*h*_s_ = *h*_c_) or the sample has the shape of a thin rod at least.
- The sample material is homogeneous and isotropic,

In addition to the listed pre-conditions, there are also other conditions that are not of interest for this study. Further approximations, for example, assume a sufficiently small change in resonant frequency [22]. Moreover, the approach according to the Equations (3) and (4) cannot be easily applied to high- and low-loss materials [33,34,35]. Nevertheless, we limit ourselves to the above-mentioned pre-conditions.

### 2.3. Evaluation for Investigations on Powders for Exhaust Gas Aftertreatment

The sample volume should be much smaller than the resonator volume for various reasons. Firstly, classical theory according to Equations (3)–(5) assumes that the electric field distribution in the resonator does not change with the insertion of the sample [22,23,24,25,30]. Since powder samples typically have a high porosity and can also be dosed flexibly in small amounts, it can be assumed, that the field disturbance caused by the introduction of the sample is negligible. Furthermore, the approach is only valid if the sample is located exactly in the electric field maximum [22,23,30]. For the presented resonator setup, the diameter of the sample is particularly critical. Especially for the higher TM_0n0_ modes, whose electric field maxima drop rapidly in radial direction, significant deviations are expected for a sample diameter of 10 mm. A better consideration of the electric field distribution inside the resonator can be achieved by a simulative approach.

Another more important aspect is that the MCP method is only applicable to the analysis of rod-shaped samples. These samples should fit through the cavity from the bottom to the top plane along the resonator axis or have at least the shape of a thin rod [24,25,31,32]. For powder samples it is conceivable to adapt the height of the filling *h*_s_ to the height of the resonator. However, the perturbation of the electric field also increases with the amount of powder. Especially for samples with high permittivity or high dissipation factor (tan*δ* = $\varepsilon^{\prime \prime }$/$\varepsilon^{\prime }$), the accuracy of the method quickly diminishes [34,35]. This fact is particularly crucial for the investigation of storage materials for exhaust gas aftertreatment (such as zeolites, ceria), since their dielectric losses increase rapidly with adsorption or reduction, and with temperature as well [19,20,21,36,37].

Since the modification of the sample diameter is not an option in this case, to reduce the quantity of the sample is recommended. However, samples with a height *h*_s_ less than the height of the cavity *h*_C_ generate a depolarization field that counteracts the excitation field and weakens the net field inside the sample [24,25,32,38,39,40]. In the literature, various approaches to describe the depolarization effect have been established using different approximations for the resonator type (rectangular, cylindrical, split-ring) and sample dimensions [32,38,39,40]. In this study, a method is presented based on theories to account for depolarization in a cylindrical resonator using the TM_0*n*0_ modes for different cylindrical sample geometries.

Furthermore, the MCP method is only suitable for the investigation of homogeneous and isotropic materials—usually solid samples [22,23,24,25]. Instead, the bulk volume of a powder consists of the volume of the powder particles and the porosity fraction of the surrounding medium (air). For powders with sufficiently small particles, however, the distribution of particles and the surrounding air can be assumed to be homogeneous. In literature, the complex permittivity of powders is most commonly described by mixing models with an effective medium approach [41,42,43]. This study provides guidance for choosing a suitable model and uses a recognized mixing model to calculate the intrinsic permittivity of solid powder particles.

For the correct determination of the complex permittivity of powder samples for exhaust aftertreatment, the field distribution of the modes, the depolarization of the sample and also the bulk density of the powder must be taken into account. The individual effects are discussed in the following.

## 3. Extension of the Microwave Cavity Perturbation Theory

### 3.1. Electric Field Distribution Inside the Cavity Resonator

**Figure 2:**
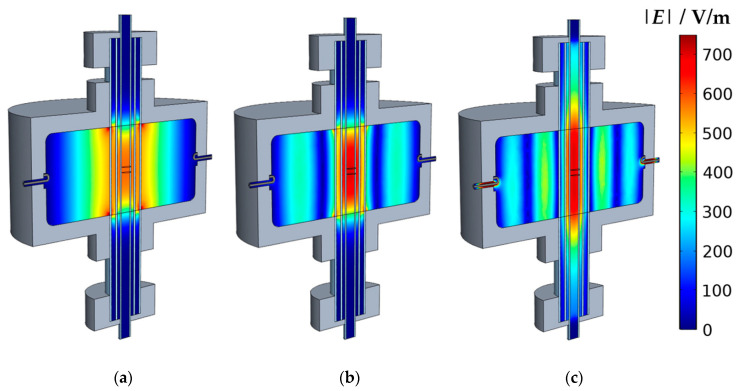
Simulated electric field inside the empty resonator (sectional view): (**a**) TM_010_; (**b**) TM_020_; (**c**) TM_030_.

The exact determination of the modal volume *V*_eff_ plays a central role for the correct evaluation of the properties of the introduced sample. The magnitude of *V*_eff_ depends mainly on the field distribution inside the cavity and, thus, also on the resonant mode. Figure 2 shows the simulated field distributions |*E*_TM_0*n*0__| of the (a) TM_010_, (b) TM_020_, and (c) TM_030_ modes. In the center of the resonator, the typical position and geometry of the sample is shown (*h*_s_ = 5 mm), whose properties match those of the filling medium (air) in this case. As it can be expected for of the TM_0*n*0_ resonant modes, the electric field maximum is located along the resonator axis. While the TM_010_ has only one maximum in the center, the higher modes have ring-shaped secondary maxima around the central axis. The width of these maxima decreases with the increasing harmonic number.

Hence, the approximation that the entire sample is located exactly in the area of the maximum electric field is doubtful, especially for the higher modes, since the electric field already decreases significantly in radial direction within the sample. This effect leads to a reduction in the RF sensitivity, or to a higher *V*_eff_, respectively. Therefore, a significant deviation from the theoretical value (simplified approach) for ideal cylindrical resonators according to Equation (5) must be expected. Instead, a more precise value for *V*_eff_ can be determined with an approach considering the electric field distribution [22,23]:

(6) $$V_{\mathrm{eff}}=V_{s}{\left(\frac{{∭}_{V_{s}}E_{0}E_{1}dV}{{∭}_{V_{c}}{|E}_{0}{|}^{2}dV}\right)}^{-1}$$

Here, the sensitivity of the RF measurement is determined by the volume integrals of the electric *E*-field squares inside the sample and the resonator volume. For small powder samples with high porosity, *E*_0_ = *E*_1_ can be assumed, since its field perturbation is negligible. The calculated values for *V*_eff_ inside the sample volume are shown in Table 1. For comparison, the values for the simplified approach for thin rod-shaped samples according to Equation (5) are also given. For the TM_010_ mode both results are obviously identical. The simplified approach may be used here, as the sample is well placed within the area of the maximum electric field (Figure 2a). The deviations between both methods (here <1%) could partially result from the accuracy of the model.

**Figure 3:**
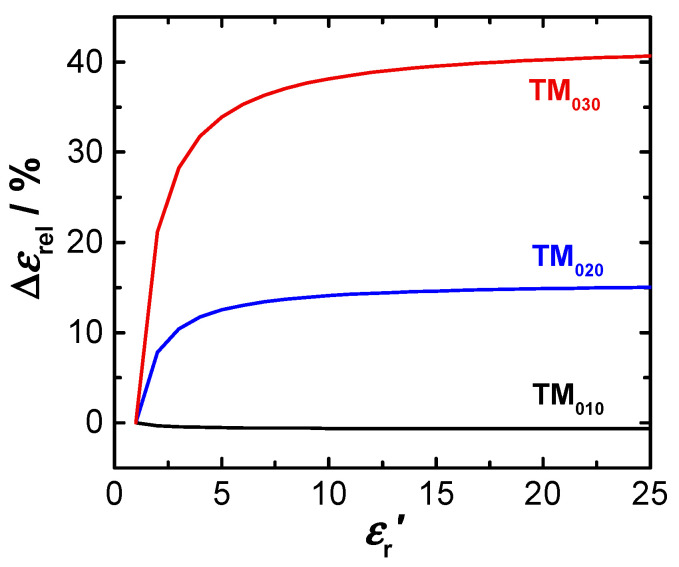
Relative deviation Δ*ε*_rel_ between both methods for the calculation of the dielectric constant as a function of the dielectric constant of the sample $\varepsilon_{r}{}^{\prime }$

However, substantial deviations between both methods are observed for the TM_020_ and TM_030_ modes ((b) and (c)). Especially for the TM_030_, the simplified approach leads to significant other RF sensitivities, or *V*_eff_, respectively. The effect is correspondingly large for the derivation of the dielectric parameters. In the case of the relative permittivity ${\varepsilon_{r}}^{\prime }$, the relative deviation Δ*ε*_rel_ between the simplified approach and the method based on the integration of electric field squares can be expressed by considering Equation (3):

(7) $${\Delta \varepsilon }_{\mathrm{rel}}=\frac{{\varepsilon_{r}}^{\prime }-{\varepsilon_{r,\mathrm{th}}}^{\prime }}{\varepsilon_{r}{}^{\prime }}=\;\frac{{(\varepsilon }_{r}{}^{\prime }-1)}{\varepsilon_{r}{}^{\prime }}\;\left(1-\frac{V_{\mathrm{eff},\mathrm{th}}}{V_{\mathrm{eff}}}\right)$$

with the actual relative permittivity of the sample $\varepsilon_{r}{}^{\prime }$, the actual modal volume $V_{\mathrm{eff}}$ in the resonator determined by the simulation (Equation (6)), as well as the modal volume $V_{\mathrm{eff},\mathrm{th}}$ (Equation (5)) and the determined dielectric constant $\varepsilon_{r,\mathrm{th}}{}^{\prime }$ according to the simplified approach. Figure 3 shows the deviation Δ*ε*_rel_ as a function of the dielectric constant $\varepsilon_{r}{}^{\prime }$ of the sample:

.

As expected, the error is negligible for the fundamental TM_010_ mode. For higher modes, the simplified calculation leads to significant errors. Already with smaller dielectric constants the deviations rapidly increase and rise (for $\varepsilon_{r}{}^{\prime }$ > 10) up to 15% in case of TM_020_ and exceed 40% for the TM_030_ mode. Hence, consideration of the real field distribution in the resonator is crucial for the correct determination of the dielectric properties in this case. For the investigation of a solid ceria sample (sintered pellet) with $\varepsilon_{r}{}^{\prime }$ = 23 [44,45,46] the approach of Equation (6) should be used. Nevertheless, even for powders whose effective dielectric constants are usually much smaller (depending on the bulk density of the powder), the error in the calculation using the simplified approach cannot be neglected. The extended approach provides a significant improvement for the evaluation of RF sensitivity when measuring a sample in the excitation field of the resonator.

At first sight, the dimension of *V*_eff_ does not depend on the sample height, since the electric field along the resonator axis remains constant for the TM_0*n*0_ modes. However, the simulation (Figure 2) shows that the electric field of all modes decreases towards the two openings at the resonator bottom and top plane. For samples with a larger *h*_s_ it is, therefore, expected that the modal volumes *V*_eff_ will increasingly differ from the ideal theoretical value. For longer samples, the method using the *E*-field squares is therefore already recommended for the TM_010_ mode. For smaller sample heights, the result for the modal volume remains widely stable, because the electric field changes along the symmetry axis can be neglected. The results shown in Table 1 and Figure 3 are therefore valid for samples with similar heights (*h*_s_ ≈ 5 mm).

For TM_030_ mode (Figure 2c), the magnitude of the electric field strength also decreases from the center along the resonator axis. Furthermore, it is noticeable that the propagation of the main maximum is not limited to the cylindrical resonant cavity, but parts of the electric field are excited outside of it along the quartz tubes. Here, an approach that considers the electric field distribution provides more precise values for the modal volume *V*_eff_. However, the accuracy of approaches for cylindrical resonators is questionable in this case.

### 3.2. Depolarization Field of the Sample

**Figure 4:**
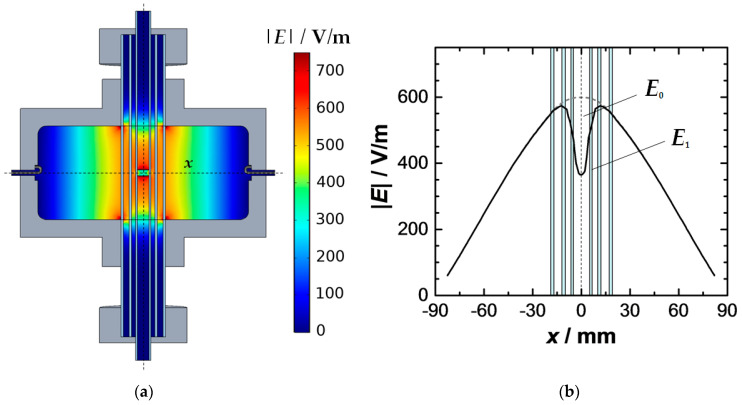
Depolarization of a ceria powder ($\varepsilon_{r,\mathrm{eff}}{}^{\prime }$ = 2.59) in the excitation field of the resonator: (**a**) electric field distribution (sectional view) in the resonator and within the sample; (**b**) electric field along the *x*-axis with pronounced weakening at the location of the sample (*x* = 0).

For samples with *h*_s_ < *h*_c_ a depolarization effect is observed within their volume. The sample material causes a depolarization field against the excitation field of the resonator. It weakens the total electric field within the sample [24,25,32,38,39,40]. For cylindrical samples, the extent of the depolarization field increases with their diameter/height ratio. The effect is, thus, more significant for flat samples than for elongated [32,40,47]. In Figure 4 the effect of depolarization is demonstrated by a simulation. The weakening of the electric field within the sample volume (*h*_s_ = 5 mm, $\varepsilon_{r,\mathrm{eff}}{}^{\prime }$ = 2.59, $\varepsilon_{r}{}^{\prime \prime }$ = 0) is clearly visible (Figure 4a). The chosen dielectric constant is typical for a loosely packed ceria powder, which will be investigated later to validate this study.

The electric field along the *x*-axis (dashed line in Figure 4a) is shown in Figure 4b. The comparison of the net field depolarized *E*_1_ (black) with the excitation field of the empty resonator *E*_0_ (grey dashed line) clearly shows the importance of the effect for the MCP method. Generally, the weakening of the electric field is also locally different. The depolarization is especially prominent in the vicinity of the upper and lower plane of the sample and decreases towards its center [40,47]. However, due to the small sample height, this local effect can hardly be observed for the selected sample shape. It should be noted that the permittivity of the sample also plays a role for the degree of depolarization. Materials with high dielectric constants yield a stronger depolarization field [40,47,48,49].

For the correct determination of the dielectric properties, a description of the sample depolarization and its effect on the RF measurement is essential. Equation (8) describes the general approach to consider the depolarization field in the sample volume [25,48]:

(8) $$E_{1}={\;E}_{0}-NP$$

with the electric (net) field within the sample *E*_1_, the excitation field *E*_0_ of the undisturbed resonator, the geometry-dependent depolarization factor *N*, and the polarization field *P* due to the introduction of the sample. The correlation between the polarization field *P* and a unidirectional excitation field *E*_0_ (here: z-axis) [38,46,49] is given by:

(9) $$P={\alpha \;E}_{0}=\;\frac{(\varepsilon -{1)E}_{0}}{1+N_{z}\;(\varepsilon -1)}$$

with the polarizability *α* and the depolarization factor *N*_z_ of the sample in *z*-direction. The depolarization factor *N* allows values between 0 and 1 and serves as a weighting factor for depolarization. Its magnitude depends on both the sample shape and the orientation of the sample in the excitation field. If the sample is polarized along all three spatial directions, the individual depolarization factors add up to a value of 1:

(10) $$N_{x}+N_{y}+N_{z}=1$$

For a sphere, the depolarization factor is 1/3 in all directions, while a very flat sample has a value close to 1, when polarized along its short axis. An elongated sample would cause almost no depolarization when polarized along its long axis and would therefore have a depolarization factor close to 0. In order to describe the depolarization within the sample properly, a correct calculation of the depolarization factor is crucial. For the presented MCP method, the powder can well be considered as a cylindrical sample polarized along its symmetry axis. However, the calculation of the depolarization factor is not trivial for cylinders. A more common method uses spheroids as an approximation, for which closed solutions exist. The assumed spheroid and the inserted sample have identical volumes. For an oblate spheroid (axes *a*, *b*, *c* with *b* = *c*), polarized along the short axis, the depolarization factor can be calculated by [47,49]:

(11) $$\;N\;=\;\frac{m^{2}}{m^{2}-1}-\frac{m^{2}}{\sqrt{{{(m}^{2}-1)}^{3}}}\mathrm{asin}\frac{\sqrt{{(m}^{2}-1)}}{m}$$

with the axis ratio *m* = *c*/*a*. Approaches also exist for prolate spheroids [47]. The method provides good approximations for *N*, especially for very flat and elongated geometries. If the height and diameter of the cylindrical sample are similar, larger deviations can still occur. In this case, a more accurate depolarization factor can be derived with a little correction for the sample polarizability [49]:

(12) $$\alpha_{\mathrm{cyl}}={\;\alpha }_{\mathrm{sph}}+\Delta \alpha$$

with the polarizability *α*_cyl_ of the cylindrical sample, the polarizability *α*_sph_ of an ellipsoid with identical axial ratio and volume and the correction term for polarizability Δ*α*. The correction term becomes small especially for very flat and very long samples. Values for Δ*α* are, for example, tabulated in the numerical study [49]. However, the described method for determining the depolarization factor does not take into account conductive surfaces near the sample. For a sample inside the cavity, a change of the depolarization factor and, therefore, of the depolarization field is expected. Parkash et al. describe the effect on the depolarization factor of the sample using the mirror charge method [25]:

(13) $$N_{e}=N\frac{{\pi h}_{S}}{{2h}_{c}}\cot\left(\frac{{\pi h}_{S}}{{2h}_{c}}\right)$$

with the effective depolarization factor *N*_e_, which still depends strongly on the sample shape and dimensions. In the resonator, however, it is also a function of the height ratio of sample *h*_s_ and resonator *h*_C_. As mentioned above, the depolarization of the sample also affects the RF sensitivity. The approach according to Equation (8) must therefore be considered in (2). The correlation between the dielectric properties of the sample and the resonant properties of the TM_0*n*0_ modes of a cylindrical resonator (filling medium: *ε*_0_, *µ*_0_) with account for sample depolarization can be described by the following equations [25]:

(14) $$\varepsilon_{r}{}^{\prime }-1=\frac{V_{c}\frac{\Delta f}{f_{0}}\left(\;\frac{V_{S}V_{C}}{{2V}_{\mathrm{eff}}}-N_{e}V_{C}\frac{\Delta f}{f_{0}}\;\right)-\frac{1}{4}N_{e}V_{C}^{\;2}{\left(\Delta \frac{1}{Q}\right)}^{2}}{{\left(\frac{V_{S}V_{C}}{{2V}_{\mathrm{eff}}}-N_{e}V_{C}\frac{\Delta f}{f_{0}}\right)}^{2}+\frac{1}{4}{\left(N_{e}V_{C}\right)}^{2}{\left(\Delta \frac{1}{Q}\right)}^{2}}$$

(15) $$\varepsilon_{r}{}^{\prime \prime }=\;\frac{\frac{1}{2}V_{c}\left(\Delta \frac{1}{Q}\right)\left(\frac{V_{S}V_{C}}{{2V}_{\mathrm{eff}}}-N_{e}V_{C}\frac{\Delta f}{f_{0}}\right)-\frac{1}{2}N_{e}V_{C}^{\;2}\frac{\Delta f}{f_{0}}\left(\Delta \frac{1}{Q}\right)}{{\left(\frac{V_{S}V_{C}}{{2V}_{\mathrm{eff}}}-N_{e}V_{C}\frac{\Delta f}{f_{0}}\right)}^{2}+\frac{1}{4}{\left(N_{e}V_{C}\right)}^{2}{\left(\Delta \frac{1}{Q}\right)}^{2}}$$

The equation is more comprehensive than the simplified correlation from Equations (3) and (4). The dielectric constant can no longer be determined solely from the change in the resonant frequency. The change of the quality factor must also be taken into account. The same applies to the dielectric loss of the sample. Parkash et al. have already shown that the sample properties for *h*_s_ < *h*_c_ can be determined much more precisely with the extended method.

To present the effect of the depolarization on the RF measurement in more detail, Equation (14) can be applied to the simulation model for the ceria powder. For this purpose, Table 2 shows the resonant properties of the filled and the empty resonator as well as the calculated effective depolarization factor and the derived dielectric constant of the sample. The resonant frequency and the quality factor of the empty resonator are provided by the simulation model shown in Figure 2. The resonant properties of the resonator with the depolarized ceria powder (*h*_s_ = 5 mm, $\varepsilon_{r,\mathrm{eff}}{}^{\prime }$ = 2.59) are determined with the model shown in Figure 4. For the sample geometry, an effective depolarization factor of 0.4354 is calculated, which meets the expectations for a slightly flat cylinder. For the modal volumes *V*_eff_ of the TM_0*n*0_ modes the simulative results of Table 1 were used.

As Table 2 shows, the results for the dielectric constant of the powder according to Equation (14) ($\varepsilon_{r,\mathrm{eff}}{}^{\prime }$ = 2.52–2.66) are closer to the actual value ($\varepsilon_{r,\mathrm{eff}}{}^{\prime }$ = 2.59). The deviation for this sample is less than 3%. The depolarization factor of 0.4354 indicates that a significant field weakening occurs within the sample and the effect must be taken into account during the MCP measurement. If the calculation were conducted with Equation (3) without considering the depolarization, the dielectric constant would strongly deviate from the actual value ($\varepsilon_{r,\mathrm{eff}}{}^{\prime }$ = 1.91–1.97). In this case, the weakened net field within the sample were ignored, which consequently would reduce the signal amplitudes in the RF measurement. As a result, the dielectric constant of the sample would be interpreted as too low.

The investigation with the simulation model, thus, confirms that depolarization effects must be considered for a correct interpretation of the data, and the Equations (14) and (15) are suitable for a precise calculation of the dielectric material properties.

### 3.3. Bulk Properties of the Powder Samples

The most common theoretical approach to describe the complex permittivity of mixtures is a dielectric mixture model. The effective permittivity of mixtures is calculated from the partial volumes and permittivity of the individual components. In the literature, many other mixture models are reported, which can lead to very different results [41,42,43,50,51,52,53,54,55,56,57,58,59,60]. Whether a mixture model is suitable for the investigation on a certain material depends on the plausibility of the derived dielectric properties [41,42,43,53,54,55,56,57,58]. In contrast to the previous sections, a general approach that allows for the reliable calculation of the sample properties independent on the powder material can therefore not be given in this study. Instead, this report is limited to the investigation of a ceria powder, as this material is typical for applications in exhaust gas aftertreatment and will be used later to validate the presented method.

**Figure 5:**
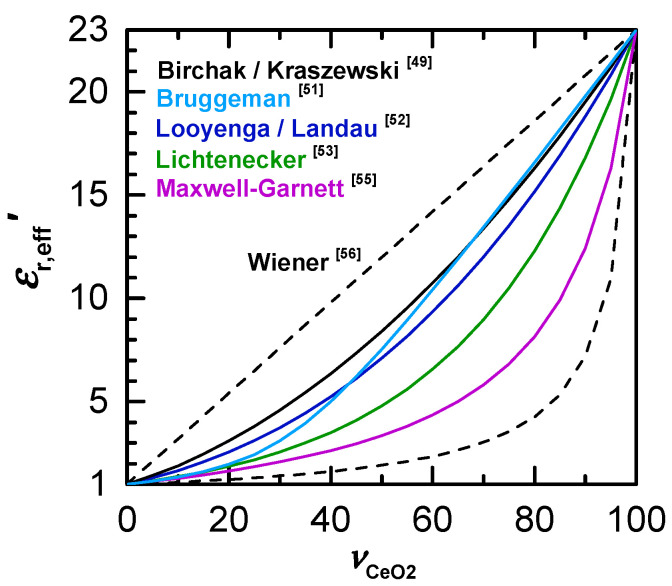
Effective dielectric constant of ceria powder, $\varepsilon_{r,\mathrm{eff}}{}^{\prime }$, as it depends on volume fraction $\nu_{\mathrm{CeO}2}$ of ceria, according to some dielectric mixing models from literature.

In the literature, a dielectric constant of $\varepsilon_{r}{}^{\prime }$ = 23 is reported for a solid ceria sample at room temperature under dry ambient conditions and frequencies in the microwave range [44,45,46]. Figure 5 shows the derived effective dielectric constant $\varepsilon_{r,\mathrm{eff}}{}^{\prime }$ of a ceria powder according to various mixing models as a function of the volume fraction $\nu_{\mathrm{CeO}2}$ of the powder. The two Wiener boundaries (dashed lines) correspond to the theoretical maximum and minimum values for the effective dielectric constant and describe the powder with a parallel and series circuit of powder material and porosity fraction. As Figure 5 infers, a wide range of possible effective properties can be derived by frequently used dielectric mixture models (Lichtenecker, Maxwell–Garnett, etc.).

Farra et al., who also applied MCP, successfully used the dielectric mixing model according to Looyenga for calculation of the dielectric properties of ceria from the powder filling [61]. The Looyenga mixing model follows the common approach [43,53]:

(16) $${{(\varepsilon }_{\mathrm{eff}})}^{k}=\overset{n}{\underset{i}{\sum }}\nu_{i}{{(\varepsilon }_{i})}^{k}$$

with the complex permittivity of the mixture *ε*_eff_, the complex permittivity *ε*_i_ and the volume fraction *ν*_i_ of the *i*-th constituent and the number of constituents *n*. For Looyenga’s model the exponent *k* is 1/3 [53]. Since the effective medium approach according to Looyenga does not take into account the shape of the particles, the formula can be used especially well for homogeneous mixtures, such as powders in this case [53]. Other studies have also shown that compared to other mixing models, Looyenga’s model gives significantly better results for volume fractions <30%, which is typical for loose powder fillings, and for strongly dissipative particles, which could be important in the chemical reduction of ceria [54,55,56]. Theoretical reflections and findings of previous studies therefore indicate that the effective medium approach according to Looyenga might be suitable for investigations on ceria powders.

**Figure 6:**
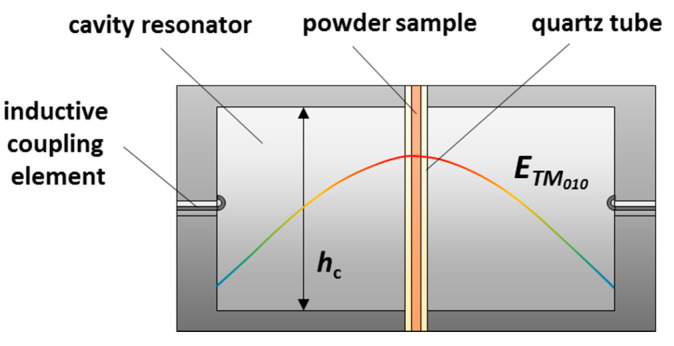
Setup for the characterization of the dielectric properties of ceria powder at room temperature.

Whether Looyenga’s law also applies for this study, can be best clarified by a comparison between measurement results and findings from literature on known material states. Therefore, the ceria powder is investigated in a simplified resonator geometry at room temperature (Figure 6). Here, the ceria powder is located in a thin quartz glass tube (Ø_a_ 5 mm, Ø_i_ 3 mm) and fills the resonator (Ø 45 mm, *h*_c_ = 40 mm) from the bottom to the top plane. To avoid water interferences, the sample was dried for 48 h at 120 °C. The volumetric proportion *ν*_CeO_2__ of ceria in the bulk is 20.6%. For the validation of a suitable mixture model, the TM_010_ mode is used, because of its superior reliability over the higher modes. For this resonator geometry, it occurs at around 2.5 GHz. The modal volume of the TM_010_ can be assumed to meet the theoretical value (26.95% of the cavity volume) in this case, since the diameter ratio between the sample and the cavity is even smaller than for the geometry of the original resonator presented in Figure 1. And since *h*_s_ = *h*_c_, obviously, any sample depolarization is avoided, and the simplified approach according to Equations (3) and (4) is valid for the given configuration.

The results for the resonant frequencies and the effective dielectric constant $\varepsilon_{r,\mathrm{eff}}{}^{\prime }$ of the powder and the ceria material are shown in Table 3. For the powder filling, $\varepsilon_{r,\mathrm{eff}}{}^{\prime }$ = 2.59 was determined, which clearly indicates that the effective dielectric constant is significantly smaller than for solid ceria ($\varepsilon_{r}{}^{\prime }$ = 23). When using Looyenga’s mixing model, a dielectric constant of 22.4 is calculated for ceria. Thus, the mixing model can be used to derive the dielectric properties, and previous studies are confirmed as well [61]. With other mixture models, the calculated properties strongly deviate from the expected values. For instance, Birchak’s or Lichtenecker’s mixing models, as listed in Table 3, provide significantly less accurate values for the dielectric constant. For other mixing rules, the deviations are even larger.

Even if Looyenga’s mixing rule provides useful results for ceria, a transfer to other materials is not easily possible. The plausibility of the mixture model must be re-checked in these cases. Zeolites and soot of exhaust gas aftertreatment systems are interesting for investigations by means of MCP as well. For these materials, other common mixture models such as those proposed by Kraszewski/Birchak [50], Bruggeman [52], Lichtenecker [57] or Maxwell–Garnett [59] could be more suitable. For the verification, however, an individual investigation is highly recommended.

## 4. Validation of the Measurement Method, Transferability and Alternative Approaches

The validation at the resonator is intended to ensure that the presented approach is suitable for determining both the dielectric constant and the dielectric loss of powder samples. In this study, a ceria powder sample (bulk density 20.6%) was investigated at room temperature and 600 °C, at which ceria acts as an oxygen storage material. This allows to investigate the oxidized and the reduced state (process gas: 21% oxygen (O_2_) in nitrogen (N_2_) and hydrogen-water mixture in N_2_ with *p*_O_2__ ≈ 10^−20^ bar, respectively). Due to the formed free electrons in the reduced state and the thermally activated mobility due to the small-polaron conduction mechanism, the conductivity of the material is strongly increased. This behavior can be well described by the defect chemistry. Details on the defect chemistry of ceria and its effects on the electrical material properties, are provided in the literature [36,37], but is beyond the scope of this work. The results for the dielectric constant and the electrical conductivity according to the TM_010_, TM_020_, and TM_030_ signals are shown in Table 4a–c.

The calculation of the dielectric properties of the sample was carried out considering the field calibration (Section 3.1), the depolarization of the sample (*N*_e_ = 0.402) (Section 3.2), and the Looyenga mixing model (Section 3.3). The results for the measurement at room temperature are given in Table 4a. For the three modes, a dielectric constant of 22.6–23.8 was determined. The calculated properties are therefore consistent with findings from literature ($\varepsilon_{r}{}^{\prime }$ = 23) [8,44,45,46]. However, the losses inside the material at room temperature are too low to be determined by the measurement method. To highlight the effects of the new approach on the MCP measurement, Table 4a also shows alternative calculations without considering the accurate electric field distribution or without considering the depolarization field. If the simplified approach according to Equation (5) is used for the field calibration, the dielectric constant can still be quite accurately determined with the TM_010_ mode ($\varepsilon_{r}{}^{\prime }$ = 23.0). This finding is in line with the results from Section 3.1. However, for the higher modes, the RF sensitivity is assumed to be higher, yielding significantly underestimated dielectric constants ($\varepsilon_{r}{}^{\prime }$ = 16.0 for the TM_020_ mode and $\varepsilon_{r}{}^{\prime }$ = 7.91 for the TM_030_ mode). This evidences that a precise consideration of the field distribution (Equation (6)) is essential for providing more accurate results for the higher modes. Similar observations can be reported if the depolarization of the sample is neglected (Ne = 0). In this case, the weakening of the net field within the sample is not considered, which effectively reduces the signal amplitudes in the RF measurement. Therefore, the determined dielectric constants are too low ($\varepsilon_{r}{}^{\prime }$ = 11.2–11.5). Since depolarization depends mainly on the shape of the sample, the phenomenon affects all three modes equally. The additional calculations show obviously that the field distribution, the occurring depolarization and the powder filling are correctly considered and a successful determination of the dielectric properties of the sample is only possible if all three effects are combined in the calculation.

Furthermore, the data in Table 4b shows that the dielectric constant of ceria ($\varepsilon_{r}{}^{\prime }$ = 17.6–22.8) remains nearly unaffected when heated to 600 °C. This observation agrees well with [8]. While almost identical values can be determined from the signals of TM010 and TM020 compared to the measurement at 25 °C, a slight decrease of the dielectric constant can be observed for the TM030 mode ($\varepsilon_{r}{}^{\prime }$ = 23.8 at 25 °C, = 17.6 at 600 °C). This deviation can be explained by the properties of the TM_030_ mode, which shows a distinct stronger attenuation compared to the other two modes. This consequently leads to smaller quality factors (<1000) and restricts the precision of the measurement. The fact that the TM_010_ and TM_020_ modes are more reliable must, therefore, be attributed to the properties of the resonator itself and not to the presented approach. Due to the small signal amplitudes for oxidized ceria, reasonable conductivities can only be calculated from the TM_010_ and TM_020_ signals.

To determine the sample conductivity, it can be assumed, that the measured losses are purely from electrons and polarization losses can be neglected ($\varepsilon_{r}{}^{\prime \prime }$ = 0). Using the MCP method, an electrical conductivity of 1.58–1.79 · 10^−5^ S/cm can be determined for the oxidized ceria at 600 °C, which again corresponds well with values measured in the literature [36]. Thus, the approach also provides plausible values for the dielectric losses of ceria.

The measurements under reduced conditions (Table 4c) also show that the reduction of ceria causes an increased sample polarization and an increased conductivity, which is again well in line with previous literature [8,36,44,45,46,47]. The calculated conductivities are very similar for all modes (4.22–5.50 · 10^−5^ S/cm) and are close to the values reported in literature [36]. The conductivity can be determined even from the TM_030_, since the signal amplitudes are significantly higher due to the reduction of the sample. The results show that the presented method allows to successfully determine the dielectric properties of the ceria sample. The polarization and dielectric loss of the sample under typical operating conditions can be evaluated properly. The presented method now takes into account the field distribution in the resonator, the depolarization of the sample and the filling of the powder. The approach extends the application range of MCP measurements significantly and provides reliable information about the dielectric properties of the inserted sample.

However, the approaches are subject to some assumptions that have to be met. It was assumed for the electric field calibration of the resonator that the excitation field E0 does not change when the sample is introduced. For sufficiently small samples, the values for the field calibration (Veff) remain identical. However, for samples with a large volume *V*_s_ or a high (effective) dielectric constant, the disturbed field has to be taken into account. This could be necessary, for example, if the bulk densities or dielectric constants of the powder particles are significantly higher. Due to the low package density of powders, the assumption should be justified in most cases. For example, the measured dielectric constant $\varepsilon_{r,\mathrm{eff}}{}^{\prime }$ for ceria was only 2.59 at a ceria volume fraction of 20.6%.

The calculation of an exact depolarization factor for the sample is much more difficult. On the one hand, the accuracy of approximation of the powder sample shape as an ideal cylinder will decreases with sample heights *h*_s_. On the other hand, errors in the estimated sample height lead to larger changes in the depolarization factor for smaller samples. In addition, Parkash et al. have already indicated that the approach according to Equations (14) and (15) can deliver inconsistent results in case of strong sample depolarization, or very flat samples, respectively [25]. In this study, the effect of the porous quartz frit on the sample depolarization was also neglected. Therefore, the height of the investigated material should preferably be as large as possible without violating other MCP conditions. Applying the method to very flat structures or materials (layers) is not recommended. It is also worth mentioning that the depolarization factor may also be determined by finite element simulation. However, this would require an individual simulation model for each investigated sample, because the simulation model must be specifically adapted to the shape and properties of the sample. An analytical approach can be implemented more quickly and also provides reasonably accurate results for the depolarization factor according to acknowledged methods. Venermo et al. quantified the deviation of their method for determining depolarization factors for cylindrical samples to be less than 1% [49]. From this perspective, the determination of the depolarization factor by simulations is also possible, but not more beneficial, since the cylindrical approximation of the sample geometry alone causes larger deviations. Alternatively, the sample depolarization can also be considered by insertion of a sample with known dielectric properties and geometry. Such calibration sample must have an identical shape as the powder sample in order to correctly determine the field weakening. However, for this method, a calibration measurement is required before each investigation, which only can be applied to the specific sample shape. An analytical approach is, hence, more flexible and can be easily adapted to the individual geometry of a sample.

However, the largest uncertainty in the evaluation of dielectric properties, likely, stems from the determination of the material properties from the effective parameters of the powder filling. Small deviations in the estimation of porosity or bulk density lead to a larger error propagation when using dielectric mixture models. In general, it can be expected that for powders with lower porosity more precise results can be obtained with the method, provided that the field disturbance by the introduction of the sample is not too large. Beyond that, the validity of a mixing model must always be checked for each material in advance using known material states under defined conditions (preferably with simple geometries).

## 5. Conclusions

In this study, a method was presented to determine the dielectric properties of powder samples using MCP. The approach allows to determine the dielectric constant and the dielectric loss of the storage material of a catalyst under typical operating conditions. Detailed reflections on the measurement method now allow to consider the electric field distribution in the resonator with a simulation model, the sample depolarization with an analytical approach and the bulk properties with a common dielectric mixing model. The method provides a significant extension to earlier publications [18,19,20,21], which served as a means of assessing the state of a typical catalyst materials (e.g., oxidized/reduced), but could not provide precise information about the electrical properties of the materials. The approach was successfully verified by measurements on a ceria powder under typical operating conditions. The method is well transferable to other materials, such as SCR and NO*_x_* storage materials or soot, with only a few modifications and, thus, has the potential to provide further important insights into microwave characteristics and the functioning of typical exhaust aftertreatment materials. The findings contribute to improving the microwave-based state diagnosis of automotive catalytic converters.

## Figures and Tables

**Table 1:** Comparison of the modal volumes of the TM_010_, TM_020_, and TM_030_ modes of the resonator.

Mode	TM_010_	TM_020_	TM_030_
***V*_eff_/*V*_C_ (simulation)**	26.78%	13.73%	12.84%
***V*_eff, th_/*V*_C_ (simplified approach)**	26.95%	11.58%	7.37%

**Table 2:** Calculated dielectric constant of a ceria powder from simulation data with and without consideration of depolarization.

Mode	*f*_s_/GHz	*f*_0_/GHz	*Q*_S_	*Q*_0_	*N*_e_	$\varepsilon_{r,\mathrm{eff}}{}^{\prime }$ (Equation (14))	$\varepsilon_{r,\mathrm{eff}}{}^{\prime }$ (Equation (13))
**TM_010_**	1.179563	1.179973	19,787	19,781	0.435	2.66	1.97
**TM_020_**	2.624186	2.625898	30,183	30,818	0.435	2.56	1.93
**TM_030_**	4.237119	4.240028	25,821	26,628	0.435	2.52	1.91

**Table 3:** Results of the investigation of the ceria powder sample in the simplified resonator geometry.

***f*_s_/GHz**	***f*_0_/GHz**	$\varepsilon_{r,\mathrm{eff}}{}^{\prime }$	$\varepsilon_{r}{}^{\prime }$ ** (Looyenga)**	$\varepsilon_{r}{}^{\prime }$ **(Birchak)**	$\varepsilon_{r}{}^{\prime }$ **(Lichtenecker)**
2.470882	2.478675	2.59	22.4	15.7	101

**Table 4:** Results for the dielectric properties of the ceria sample inside the resonator cavity: (a) measured at 25 °C and 21% O_2_; (b) at 600 °C and 21% O_2_; (c) at 600 °C and *p*_O2_ = 10^−20^ bar. The derived values for the material permittivity are highlighted. Please note the lower permittivity for higher modes when applying simplifications. The permittivity for the TM_030_ mode in (b) is written in brackets, due to its uncertainty.

**(a) Conditions: 25 °C, *p*_O2_ = 0.21 bar**				**Literature:** $\varepsilon_{r}{}^{\prime }$ **= 23 [44,45,46]**				
**Mode**	***f*_s_/GHz**	***f*_0_/GHz**	***Q*_S_**	***Q*_0_**	***N*_e_**	***V*_eff_/*V*_C_**	$\varepsilon_{r}{}^{\prime }$	***σ*/(S/cm)**
**TM_010_**	1.180624	1.180870	13,890	13,930	0.402	26.78%	22.6	--
**TM_020_**	2.629937	2.631025	13015	12,756	0.402	13.73%	23.6	--
**TM_030_**	4.200389	4.203252	893.34	898.65	0.402	12.84%	23.8	--
**For comparison: Calculation with simplified electric field calibration (*V*_eff_):**								
**TM_010_**	1.180624	1.180870	13,890	13,930	0.402	26.95%	23.0	--
**TM_020_**	2.629937	2.631025	13015	12,756	0.402	11.58%	16.0	--
**TM_030_**	4.200389	4.203252	893.34	898.65	0.402	7.37%	7.91	--
**For comparison: Calculation without considering depolarization (*N*_e_ = 0):**								
**TM_010_**	1.180624	1.180870	13,890	13930	0	26.78%	11.2	--
**TM_020_**	2.629937	2.631025	13,015	12756	0	13.73%	11.5	--
**TM_030_**	4.200389	4.203252	893.34	898.65	0	12.84%	11.5	--

**(b) Conditions: 600 °C, *p*_O2_ = 0.21 bar**				**Literature:** $\varepsilon_{r}{}^{\prime }$ **= 23 [8,43,44,45,46], *σ* = 3.9 10^−5^ S/cm [36]**				
**Mode**	***f*_s_/GHz**	***f*_0_/GHz**	***Q*_S_**	***Q*_0_**	***N*_e_**	***V*_eff_/*V*_C_**	$\varepsilon_{r}{}^{\prime }$	***σ*/(S/cm)**
**TM_010_**	1.179082	1.179328	12,327	12826	0.402	26.78%	22.7	1.79·10^−5^
**TM_020_**	2.625354	2.626425	9572.3	10341	0.402	13.73%	22.8	1.58·10^−5^
**TM_030_**	4.192808	4.194524	943.23	1210.1	0.402	12.84%	<17.6>	--

**(c) Conditions: 600 °C, *p*_O2_ = 10^−20^ bar**				**Literature: *σ* = 2.0 10^−2^ S/cm [36]**				
**Mode**	***f*_s_/GHz**	***f*_0_/GHz**	***Q*_S_**	***Q*_0_**	***N*_e_**	***V*_eff_/*V*_C_**	$\varepsilon_{r}{}^{\prime }$	***σ*/(S/cm)**
**TM_010_**	1.178967	1.179341	3441.6	12,878	0.402	26.78%	42.8	4.22 10^−2^
**TM_020_**	2.625034	2.626473	2488.3	10,335	0.402	13.73%	39.0	5.50 10^−2^
**TM_030_**	4.192956	4.194998	806.22	1210.8	0.402	12.84%	23.5	5.48 10^−2^
